# Metabolic alterations in Parkinson’s disease astrocytes

**DOI:** 10.1038/s41598-020-71329-8

**Published:** 2020-09-02

**Authors:** Tuuli-Maria Sonninen, Riikka H. Hämäläinen, Marja Koskuvi, Minna Oksanen, Anastasia Shakirzyanova, Sara Wojciechowski, Katja Puttonen, Nikolay Naumenko, Gundars Goldsteins, Nihay Laham-Karam, Marko Lehtonen, Pasi Tavi, Jari Koistinaho, Šárka Lehtonen

**Affiliations:** 1 A.I.Virtanen Institute for Molecular Sciences, University of Eastern Finland, Neulaniementie 2, 70211 Kuopio, Finland; 2 Neuroscience Center, University of Helsinki, Haartmaninkatu 8, 00014 Helsinki, Finland; 3 School of Pharmacy, University of Eastern Finland, Kuopio, Finland; 4 LC-MS Metabolomics Center, Biocenter Kuopio, Kuopio, Finland

**Keywords:** Neuroscience, Stem cells

## Abstract

In Parkinson`s disease (PD), the loss of dopaminergic (DA) neurons in the substantia nigra pars compacta is associated with Lewy bodies arising from the accumulation of alpha-synuclein protein which leads ultimately to movement impairment. While PD has been considered a disease of the DA neurons, a glial contribution, in particular that of astrocytes, in PD pathogenesis is starting to be uncovered. Here, we report findings from astrocytes derived from induced pluripotent stem cells of *LRRK2* G2019S mutant patients, with one patient also carrying a *GBA* N370S mutation, as well as healthy individuals. The PD patient astrocytes manifest the hallmarks of the disease pathology including increased expression of alpha-synuclein. This has detrimental consequences, resulting in altered metabolism, disturbed Ca^2+^ homeostasis and increased release of cytokines upon inflammatory stimulation. Furthermore, PD astroglial cells manifest increased levels of polyamines and polyamine precursors while lysophosphatidylethanolamine levels are decreased, both of these changes have been reported also in PD brain. Collectively, these data reveal an important role for astrocytes in PD pathology and highlight the potential of iPSC-derived cells in disease modeling and drug discovery.

## Introduction

Parkinson’s disease (PD) affects more than 6 million people worldwide making PD the second most common neurodegenerative disease^1^. The exact cause for PD is still unknown, but several molecular mechanisms have been identified in PD pathology. These include neuroinflammation, mitochondrial dysfunction, dysfunctional protein degradation and alpha-synuclein (α-synuclein) pathology^2^. The major hallmarks of the disease comprise the loss of dopaminergic (DA) neurons in substantia nigra (SN) pars-compacta and the presence of Lewy bodies and Lewy neurites in the patients brain. The loss of DA neurons and the subsequent decrease in dopamine levels in the striatum are considered responsible for the typical movement symptoms (bradykinesia, rigidity, rest tremor and postural instability) seen in PD^3^. There is no cure for PD and currently the treatments are targeted to alleviate the motor symptoms with dopamine replacement therapy and surgery.

The greatest risk factor for PD is high age but some environmental factors, such as toxins and pesticides have been shown to increase the risk for PD. Though most cases of PD are late onset and sporadic with no evidence for inheritance, approximately 3–5% of the cases are monogenic and 20 PD-associated genes have been identified to date^4^. The most common cause for monogenic PD are mutations in the leucine-rich repeat kinase 2 (*LRRK2*) gene. LRRK2-associated familial PD is clinically closest to sporadic forms of the disease regarding the age of onset, disease progression and motor symptoms. *LRRK2* mutations, in particular G2019S, are of great interest due to their high incidence, as they constitute more than 10% of the familial PD cases. DA neurons differentiated from induced pluripotent stem cells (iPSCs) that were derived from PD patients with *LRRK2* mutations, manifest sensitivity to oxidative stress^5^, reduced outgrowth of neuronal processes^6,7^ increased susceptibility to neurotoxins and autophagy deficits^6^. Despite intensive research efforts, it is still unknown why *LRRK2* defects specifically impair the survival of DA neurons. Mutations in the *GBA* (glucosylceramidase beta) gene are the most significant risk factor for PD identified to date. The molecular mechanisms by which GBA mutations result in this increased risk are currently the focus of substantial research efforts. Studies using iPSC-derived midbrain DA neurons from PD patients harboring *GBA* N370S mutation showed a reduction in glucocerebrosidase (GCase) activity, increased α-synuclein levels and a reduced capacity to synthesize and release dopamine^8^. The loss of GCase activity results in impairment of the autophagy-lysosome pathway^9^, which may be related to the accumulation of α-synuclein.

The yet unresolved pathophysiology of PD has driven the need for better disease models. Although animal models and toxicity-based in vitro studies have been instrumental in defining PD etiology, they can never completely recapitulate the human disease. Among the novel technologies to study neurodegenerative diseases including PD is the use of stem cell reprogramming. This technology is especially suitable for investigating diseases from which affected tissues are not easily accessible prior to autopsy. While studies focused on DA neurons have brought new insight into PD pathology^6,8–11^, astrocyte contribution to PD has been investigated only sparsely. Astrocytes are glial cells and the most abundant cell type in the human brain^12^. It was long thought that the astrocytes worked solely as supporting cells for neurons, but today the role of astrocytes is known to be far more extensive. Until now, only a few studies have used iPSC-derived astrocytes obtained from PD patients. These studies showed dysfunction in the clearance of α-synuclein^13^ and downregulation of *MMP2* and *TGFB* genes^14^ in *LRRK2* G2019S mutant astrocytes, and low glucocerebrosidase activity with accumulation of glucosylceramide in *GBA* N370S astrocytes^15^. The accumulation of astrocyte-derived α-synuclein correlated with the decreased number of DA when *LRRK2* G2019S mutant astrocytes were co-cultured with healthy DA neurons. As this study proposed an astrocytic contribution to DA cell death in PD, we generated iPSC-derived astrocytes from two PD patients carrying the G2019S mutation in the *LRRK2* gene, one of them presenting with an additional mutation in *GBA* (p.N370S)^16^ to further characterize the PD astrocyte phenotype.

## Results

### Differentiation and characterization of human iPSC derived PD astrocytes

**Figure 1:**
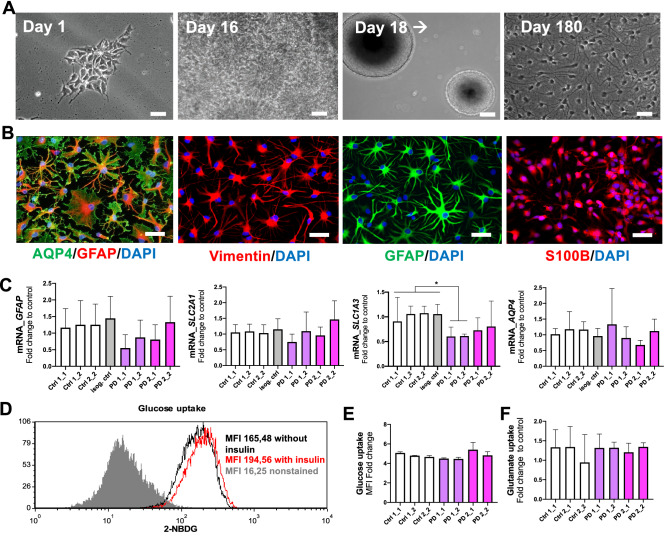
Differentiation and characterization of hiPSC-derived astrocytes from healthy subjects and PD patients. (**A**) Representative images from astrocyte differentiation. Bright field images showing hiPSCs at day 1, the rosettes at day 16, progenitor cells expanded as spheres from day 18 onwards and matured astrocytes at day 180. (**B**) Representative fluorescence images of astrocytes matured for 7 days and stained for AQP4, Vimentin, GFAP and S100B. Nuclei are stained with DAPI. Scale bars, 50 µm. (**C**) Relative gene expression levels *GFAP, SLC2A1, SLC1A3* and *AQP4* in astrocytes shown as fold change from healthy control. (**D**) Representative FACS histogram of glucose uptake analysed by fluorescent glucose analog 2-NBDG. Grey area shows untreated cells, black line shows cells incubated with 2-NBDG and red line shows cells incubated with insulin and 2-NBDG. (**E**) Glucose uptake analyzed by fluorescent glucose analog 2-NBDG. (**F**) Glutamate uptake measured with radiolabeled glutamic acid. Three independent experiments. Bars represents mean ± SD, *p < 0.05. PD1 patient carries a mutation in LRRK2 (G2019S) and GBA (N370S), PD2 patient carries a mutation in LRRK2 (G2019S) and isogenic control for PD2 patient.

We adopted a previously published method to generate astrocytes from human iPSCs^17,18^. In this method, astroglial progenitors were first generated via neuroepithelial interphase and cultured as spheres before differentiation to astrocytes (Fig. 1A). The astrocytes were maturated by dissociating the spheres and plating in astro-maturation medium for 7 days. At the age of 6 months, more than 90% of the maturated cells expressed glial fibrillary protein (GFAP), S100 Calcium-binding protein beta (S100ß), Vimentin and aquaporin 4 (AQP4) and acquired star-like morphology (Fig. 1B). In addition, the mRNA expression levels of astrocyte related markers including *AQP4* and *GFAP* and transporters GLUT1 (*SLC2A1)* and GLAST (*SLC1A3)* were examined (Fig. 1C). While *GLAST* showed higher expression level in healthy and isogenic control cells than in PD cells, the levels of *GLUT1, GFAP* and *AQP4* were unchanged between the studied groups (Fig. 1C). No evident differences were observed in glucose or glutamate uptake between the genotypes (Fig. 1D–F). Overall, all iPSC lines were able to generate comparable and functional astrocytes.

### Increased expression of alpha-synuclein in PD astrocytes

**Figure 2:**
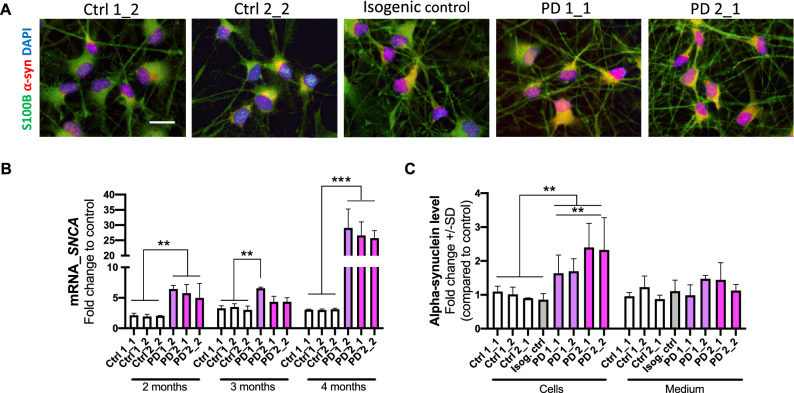
Alpha-synuclein protein expression is increased in LRRK2 mutant astrocytes. (**A**) Representative immunofluorescent images of astrocytes stained for α-synuclein and S100B. Nuclei stained with DAPI. Scale bar 20 µm. (**B**) Relative gene expression level of *SNCA* gene in astrocytes over 4-month period shown as fold change to healthy cells. The results are normalized to beta-actin expression. (**C**) Protein expression in astrocytes and the released amount of α-synuclein to media measured with ELISA. Data is shown as a fold change to control (healthy) and results are normalized to total protein content. Four independent experiments. Bars represents mean ± SD, **p < 0.01, ***p < 0.001.

The expression of α-synuclein in astrocytes was detected with ICC and quantitated by qRT-PCR for mRNA transcript and ELISA for protein levels. The mRNA expression level of *SNCA,* the gene encoding α-synuclein—a small protein abundant in brain in the presynaptic terminals of neurons, was increased in PD astrocytes at all studied time points over four-month period (Fig. 2B). This increase was also translated to protein levels, which were significantly higher in the PD astrocytes than in healthy and isogenic control astrocytes at the age of 6 months (Fig. 2A,C). The α-synuclein secreted from the astrocytes to the medium did not show differences between the studied groups (Fig. 2C).

### PD astrocytes manifest a reactive phenotype with increased cytokine secretion after inflammatory stimulation

**Figure 3:**
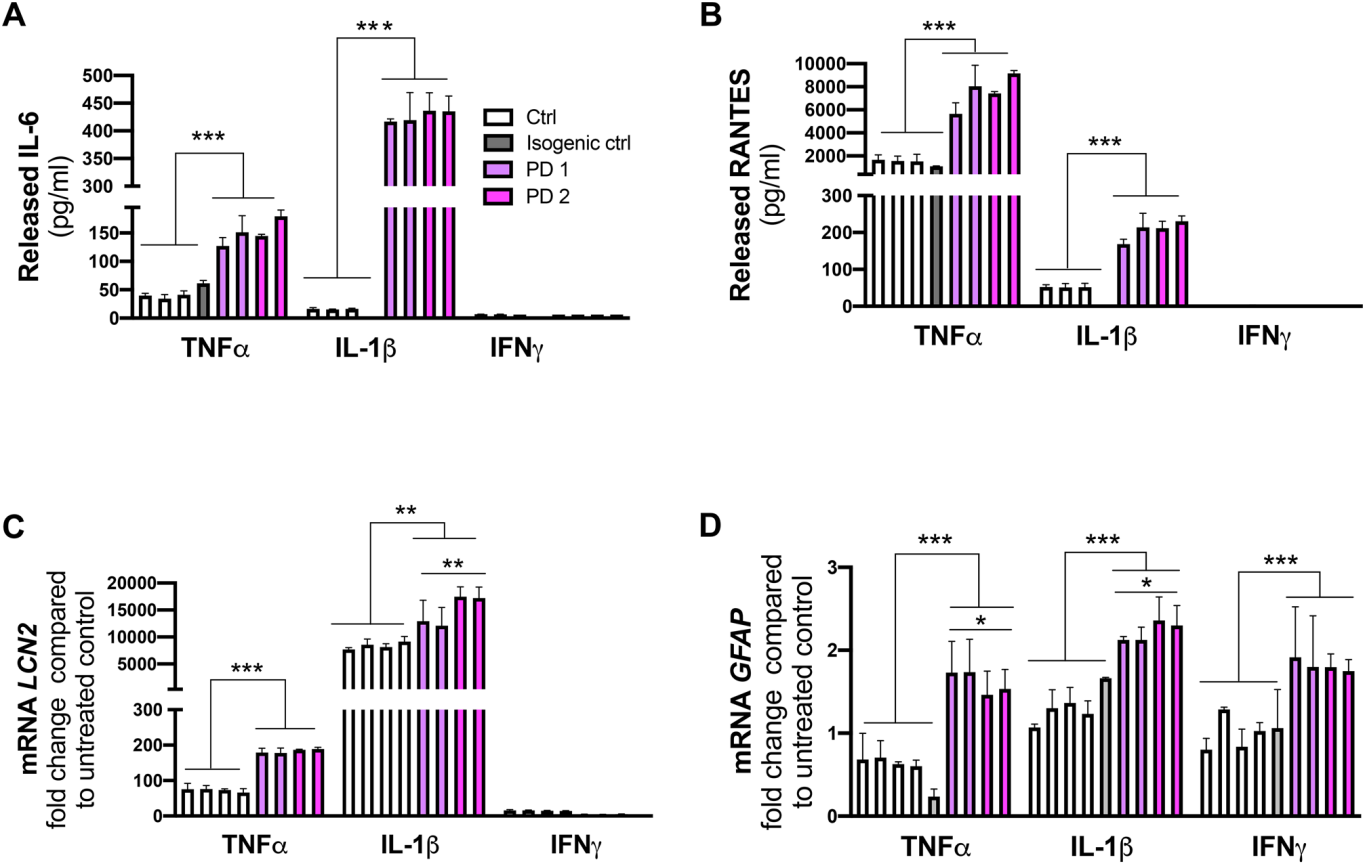
PD astrocytes showed altered phenotype in pro-inflammatory conditions. Release of (**A**) IL-6 and (**B**) RANTES quantified from media after TNFα (50 ng/ml), IL-1β (10 ng/ml) and INFγ (10 ng/ml) stimulation for 24 h measured with CBA assay. Relative gene expression levels of (**C**) *LCN2* and (**D**) *GFAP* in astrocytes treated with TNFα, IL-1β and INFγ shown as fold change from untreated healthy control. Results are presented as mean ± SD from three independent experiments, *p < 0.05, **p < 0.01, ***p < 0.001. The control lines used in the measurements (**A**) and (**B**): Ctrl 1_1, Ctrl 1_2 and Ctrl 2_2.

To assess the effect of inflammatory stimulation on secretion of cytokines from astrocytes, we first exposed the cells to TNFα, IL-1β and IFNγ. IFNγ exposed cells secreted neither IL-6 nor RANTES which is in line with previous knowledge that IFNγ activates microglia rather than astrocytes (Fig. 3A,B). TNFα and IL-1β significantly increased secretion of both IL-6 and RANTES from PD astrocytes when compared to control cells and isogenic control. We further investigated the gene expression of *GFAP* and *LCN2* (lipocalin-2) in the same inflammatory stimulating conditions. As expected, inflammatory stimulation increased mRNA expression of both genes that are associated with reactive stage of astrocytes. However, the PD astrocytes expressed significantly more *GFAP* and *LCN2* than control cells when stimulated with TNFα or IL-1β (Fig. 3C,D). Taken together, these data suggest that PD astrocytes are more responsive to inflammatory stimuli than control cells and that lipocalin-2, a known mediator of reactive astrocytosis^19^ may play a pathogenic role in PD.

### Disturbed calcium signaling in PD astrocytes

**Figure 4:**
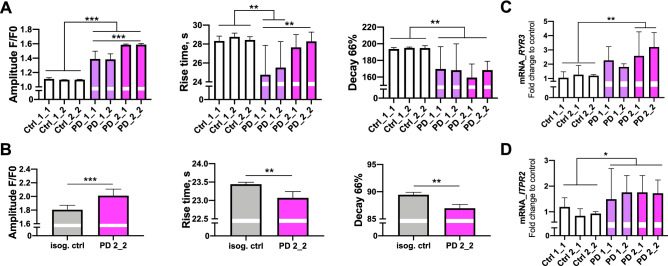
Disturbed calcium signaling in endoplasmic reticulum of PD astrocytes. The [Ca^2+^]_i_ signals in healthy, PD and isogenic control-derived astrocytes. (**A**) The amplitude F/F_0_, rise time and decay time in seconds (66%) of Ca-responses caused by ryanodine receptors activation (stimulation with cresol) in healthy and PD astrocytes. (**B**) Repeated experiment with isogenic control and PD astrocytes showing that the *G2019S* mutation in LRRK2 is responsible for the calcium disturbances in PD astrocytes, n = 800–1,200 cells. Bars represents mean ± SEM. Relative gene expression levels of ER calcium-channels (**C**) *RYR3* and (**D**) *ITPR2* (inositol 1,4,5-triphosphate receptor type 2) in astrocytes shown as fold change from healthy control. Results are presented as mean ± SD from three independent experiments, *p < 0.05, **p < 0.01, ***p < 0.001.

Given that *LRRK2* G2019S mutations are associated with controlling ER calcium, we next assessed intracellular and ER-specific Ca^2+^-homeostasis. Ca^2+^ release was stimulated by RyR agonist Cresol. First, we compared Ca^2+^ signaling between PD mutant and healthy control astrocytes (Fig. 4A; n = 7,824 PD cells vs 884 control cells). The amplitude of [Ca^2+^]_i_ responses to 4-µ-Cresol application was higher in PD astrocytes when compared to healthy counterparts. Moreover, the signal rise and decay times were shorter in PD astrocytes. Similar alterations in Ca^2+^ responses were observed when comparing *LRRK2* mutant astrocytes (PD2) to the patient cells where the mutation was corrected (Fig. 4B; n = 2,123 PD cells vs n = 1792 isogenic control). The amplitude was higher and both rise time and decay time were shorter in *LRRK2* mutant astrocytes, suggesting that the changes were a direct consequence of the *LRRK2* mutation. As the faster Ca^2+^ responses suggested alterations in ER Ca^2+^ channels, we analyzed the expression of RyRs and *ITPR2* and found that the mRNA levels of *RYR3* were increased in PD2 cells while *ITPR2* in both PD when compared to controls (Fig. 4C,D). The higher amplitude of Ca^2+^ signals in PD astrocytes indicates increased level of ER loading with Ca^2+^ and increased amount of the available Ca^2+^ whereas the faster rise of [Ca^2+^]_i_ responses corresponds with the upregulation of RyRs in PD cells (Fig. 4C).

### Altered metabolism in PD astrocytes

**Figure 5:**
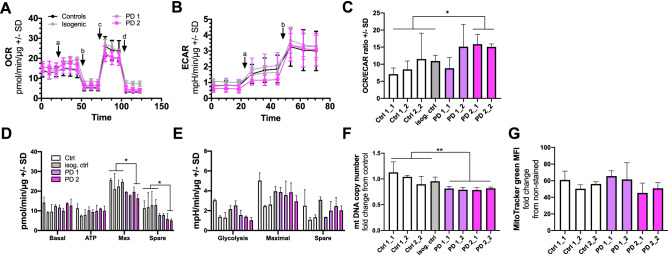
PD astrocytes have altered metabolic profile. (**A**) Oxygen consumption rate (OCR) following additions of 10 μM glucose (**A**), 1 μM oligomycin (**B**), 1 μM FCCP (**C**), and 1 μM antimycin A and rotenone (**D**) in astrocytes. (**B**) Extracellular acidification rate (ECAR) following additions of 10 μM glucose (**A**) and 1 μM oligomycin (**B**). Results are normalized to protein content and shown as mean ± SD from three independent experiments. (**C**) The OCR/ECAR ratio calculated after glucose addition. (**D**) Calculated OCR values of basal respiration, ATP production, maximal respiration, and spare respiratory capacity. (**E**) Calculated ECAR values of glycolysis, maximal glycolysis, and spare glycolytic capacity. (**F**) mtDNA copy number measured from astrocytes. Results are shown as relative fold change to healthy cells. (**G**) MitoTracker green Mean Fluorescence Intesity (MFI) per cell, representing mitochondrial mass. Bars represents mean ± SD, *p < 0.05, **p < 0.01. The order of lines in (**D**) and (**E**) is the same as in (**C**).

We next analyzed the metabolic activity of the cells by Seahorse XF Technology. First, we measured oxygen consumption rate (OCR; Fig. 5A) and extracellular acidification rate (ECAR; Fig. 5B) of the astrocytes and calculated the ratio of OCR/ECAR (Fig. 5C). While no difference was seen in basal levels, the maximal and spare respiration were significantly decreased in the PD2 astrocytes indicating altered mitochondrial function in PD2 astrocytes (Fig. 5D). The same trend was also seen with the ECAR levels with the exception that the decreased ECAR levels already appeared at the basal stage, and that the differences of maximal and spare glycolysis between PD and healthy astrocytes were only moderate (Fig. 5E). In the basal level, the healthy astrocytes were more glycolytic as typical for astrocytes when compared to the PD cells. Because dysregulation of mitochondrial DNA (mtDNA) homeostasis is known to contribute to the pathogenesis in PD^20^, we investigated mtDNA copy number from our cells. Astrocytes obtained from PD patients contained significantly lower levels of mtDNA with no change in mitochondrial mass when compared to the astrocytes from healthy controls (Fig. 5F,G). Thus, impaired mtDNA maintenance could lead to reduced mitochondrial respiration in astrocytes and mitochondrial dysfunction that is observed in both PD-patients with *LRKK2*^21^ and *GBA* mutations^22^.

### Non-targeted metabolite profiling

**Figure 6:**
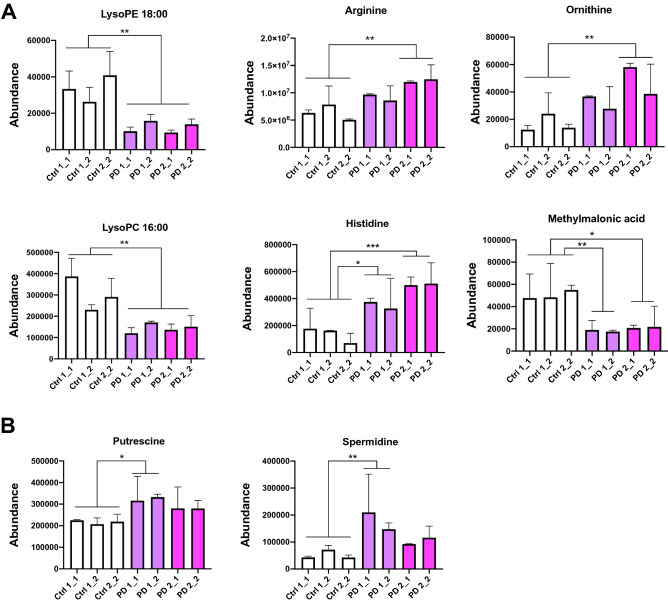
Non-targeted metabolite profiling of astrocytes revelaled several statistically significantly differentiating compounds in cell lysates (**A**) and media (**B**) of healthy and PD astrocytes analyzed by UHPLC-QTOF-MS. Bars represents mean ± SD, *p < 0.05, **p < 0.01, ***p < 0.001. *LysoPE* lysophosphatidylethanolamine, *LysoPC* lysophosphatidylcholine.

We used product ion spectrum to identify statistically significant metabolites from all molecular features. The total number of molecular features identified from cells and media are summarized in tables showing the impact of p-value (Supplementary Table S5, S6). PD astrocyte pathology was associated with alterations in the levels of multiple metabolites compared to the control astrocytes (Supplementary Table S7). These include significantly increased levels of polyamines putrescine and spermidine in PD1 astrocytes and their precursors arginine, ornithine in PD2 astrocytes (Fig. 6A,B). On the contrary, PD astrocytes manifested decrease in several metabolites including phospholipids (lysophosphatidyl-ethanolamines and -cholines; lysoPE and lysoPC respectively), amino acids and methylmalonic acid (Fig. 6A,B). Altered polyamine^23^ and phospholipids^24^ levels have been previously linked to Parkinsons and our results show that the PD astrocytes manifest these known metabolic alterations.

## Discussion

While PD has been considered as a disease of the DA neurons, it is becoming more and more evident that other cell types of the CNS, including astrocytes, play an important role in PD pathogenesis. Several genes known to have a causative role in the development of PD, such as *PARK2*, *PARK7*, *PINK1*, *LRRK2*, *SNCA*, *ATP13A2*, *PLA2G6*, *GBA* are highly expressed in astrocytes and play important roles in astrocytic functions including in the inflammatory response, glutamate uptake, mitochondrial function, lysosomal function, autophagy, oxidative stress, calcium signaling, neurotrophic capacity and neuroprotection^25^. A recent in vitro study showed that PD astrocytes manifest dysfunction in the clearance of α-synuclein^13^. When LRRK2 mutant iPSC-derived patient astrocytes were co-cultured with healthy DA neurons, the accumulation of astrocyte-derived α-synuclein correlated with a decreased number of DA neurons, suggesting astrocytic contribution to DA neuron survival^13^. In GBA mutant iPSC-derived patient astrocytes endogeneous α-synuclein was not expressed but upon exposure to exogeneous α-synuclein monomers or fibrils, the reduced GCase activity led to α-synuclein accumulation in the astrocytes^15^. Therefore, we wanted to further characterize the astrocyte phenotype in PD patients and report here that *LRRK2* G2019S and *GBA* N370S mutant PD patient astrocytes manifest several hallmarks of the disease seen in PD patient brains, including increased reactivity to inflammatory stimulation, increased Ca^2+^ release from ER and altered polyamine metabolism.

One of the main hallmarks of PD pathology is the accumulation of α-synuclein in the patient`s brain. Postmortem PD patient brain samples have revealed α-synuclein accumulation in astrocytes^26^. Furthermore, it has been previously described that astrocytes accumulate neuron-derived α-synuclein as a mechanism of neuroprotection^25^. In this study, we found that in the absence of neurons the iPSC-derived PD astrocytes themselves produced more α-synuclein that the control cells. As one of the key pathological features caused by α-synuclein aggregation is the disruption of Ca^2+^ homeostasis^27^, and LRRK2 is known to have a direct effect on calcium signaling^28^, we further looked at Ca^2+^ levels in PD astrocytes and found them to be increased. It is well known that excess levels of Ca^2+^ in brain cells may lead to the formatin of toxic clusters that are hallmarks of PD and that human brain tissue with *GBA1* mutations show enhanced Ca^2+^ release via RyRs^29^. However, iPSC-derived DA neurons carrying *LRRK2* G2019S mutation showed decreased ER Ca^2+^ levels^30^. Our results of *LRRK2* and *GBA* mutant astrocytes showing increased Ca^2+^ release from ER via RyRs, suggests that astrocytes contribute to the increased Ca^2+^ levels in PD patients brain.

Inflammation is considered to be an important contributor to PD pathology together with the presence of reactive astrocytes in the SN of PD patients^31^. Inflammatory responses manifested by glial reactions and increased expression of inflammatory cytokines and other toxic mediators derived from activated glial cells are currently recognized as prominent features of PD^32^. Studies with animal models have suggested that inflammation may drive the progressive loss of SN DA neurons. Recent in vitro studies, however, have suggested that the inflammatory activation of microglia and astrocytes may take place via mediators released by injured DA neurons, thus suggesting that the inflammatory response would be secondary to the DA cell death^32^. We show here that the PD patient astrocytes are highly responsive to inflammatory stimuli and more sensitive to inflammatory reactivation than control astrocytes. They secrete more cytokines than the control cells and moreover, the expression of *LCN2*, a molecule secreted by reactive astrocytes under inflammatory conditions^19^, is increased in PD astrocytes when compared to control cells. LCN2 regulates diverse cellular processes such as cell death/survival, cell migration/invasion, cell differentiation, iron delivery, inflammation, insulin resistance, and tissue regeneration. It promotes apoptosis, morphological changes, and migration in astrocytes both in vitro and in vivo. Activated astrocytes release LCN2 not only to induce the morphological transformation associated with reactive astrocytosis but also to promote their own death. A recent animal study with *LCN2* defective mice suggests that LCN2 is a neuroprotective agent and protects the brain upon inflammatory conditions^33^.

Mitochondrial defects are thought to be involved in PD pathogenesis. Several Parkinson-inducing toxins inhibit complex I (CI) of the mitochondrial respiratory chain and CI inhibitors, such as rotenone, have been shown to specifically target DA neurons^34^. Interestingly, MPTP (1-methyl-4-phenyl-1,2,3,6-tetrahydropyridine) toxicity to DA neurons is astrocyte-mediated. Rodent astrocytes take up MPTP, convert it to the toxic MPP+ and release it to the extracellular space from where dopamine transporters preferentially absorb the toxin^35^. These results suggest that mitochondrial respiration and especially NADH oxidation is critical for DA neurons and explain why mitochondrial defects can induce parkinsonism, a clinical syndrome characterized by movement disorders commonly seen in PD. Several subunits of the CI are encoded by mtDNA and known mtDNA defects often manifest as CI deficiency. Thus the maintenance of the mitochondrial genome is important for the survival of DA neurons. This is evident as mutations in POLG, the DNA polymerase that replicates mtDNA, are known to induce parkinsonism^36^. High levels of mtDNA deletions have been detected in the SN neurons of deceased PD subjects, and these are also present in aged subjects^37^. An intensive study of mtDNA in the DA SN neurons of healthy and PD individuals further revealed that in healthy individuals, the mtDNA copy number increases in DA SN neurons with age, maintaining the pool of wild-type mtDNA population in spite of accumulating deletions. However, in PD patients this compensatory upregulation fails and as a result the full-length mtDNA population becomes depleted. These results suggest that dysregulated mtDNA homeostasis is a key process in the pathogenesis of neuronal loss in PD^20^. We show here that mtDNA copy number is dysregulated also in PD astrocytes, suggesting that mtDNA maintenance defects may be present also in CNS cell types other than DA neurons. Since mtDNA abnormalities in the SN are associated with respiratory chain dysfunction^38^, we next checked mitochondrial respiration in PD astrocytes. Interestingly, *LRRK2* and *GBA* mutations decreased the maximal respiration capacity of astrocytes while mitochondrial mass stayed unchanged, suggesting mitochondrial dysfunction in PD astrocytes.

The endogenous polyamines (spermine, spermidine, and putrescine) are present at relatively high concentrations in the mammalian brain and play crucial roles in a variety of functional aspects, including cell proliferation and differentiation, gene transcription and translation, modulation of the functional activity of ion channels and receptors as well as several other vital processes. Polyamine concentrations have been shown to be elevated in the CSF of PD patients and polyamine contents have been suggested to serve as a metabolic marker for the diagnosis of PD^39^. The increased polyamine levels may contribute to PD pathogenesis, as spermine is known to regulate the binding of dopamine to vesicles in DA neurons^40^. Further, polyamines have been shown to enhance α-synuclein toxicity and increase α-synuclein aggregation^41^. Inflammation and stress can stimulate polyamine accumulation in glia, elevating their levels and the levels of their breakdown products^42^ and prolonged elevation of polyamine levels has been suggested to lead to gliosis^23^. We detected increased secretion of putrescine and spermidine to the media from the PD astrocytes as well as increased polyamine precursor, arginine and ornithine, levels in the astrocytes themselves without any inflammatory stimulation, suggesting that either the PD astrocytes manifest an innate defect in polyamine metabolism or some cell intrinsic stress response is stimulating polyamine production in PD astrocytes.

The phospholipids, like phosphatidylethanolamine (PE), have roles in regulating cellular proliferation, metabolism and stress responses, and are found in biological membranes particularly in nervous tissue. Normal PE levels in the brain can decline with age. PE levels in the SN of PD patients are lower than in control subjects^43^ and the levels are also significantly reduced in the CSF of PD patients^44^. Likewise, we detected a significant decrease in lysoPE levels in PD astrocytes when compared to healthy control cells. This fits well with the data from Di Domenico et al. showing that the G2019S *LRRK2* mutant PD patient astrocytes manifest autophagy defects, as PE is essential for the synthesis of glycosylphosphatidylinositol-anchored protein, which triggers autophagosome formation^13^. Further, worm studies have shown that low PE levels lead to decreased mitochondrial respiration and accumulation of α-synuclein^45^, both also present in our PD astrocytes.

PD is a multifactorial disease with various environmental and genetic factors underlying the disease progression in the patients. While the disease mechanisms may not be completely identical in all PD patients, some common mechanisms are seen in all of them. As many iPSC-based studies, also our study is limited with the number of patients and cell lines available for the study and thus our results may not represent the situation in all PD patients, but rather those with *LRRK2* mutations. The *GBA* mutation present in one of the patients, may induce variation between the patients. However, the lines from the two patients behaved very similarly in most of the experiments, suggesting that for the aspects reported here, the *GBA* mutation does not induce significant additional effects apart from those induced by the *LRRK2* mutation. Further, the reversal of the phenotype in the corrected isogenic control line verifies that the reported changes are indeed due to the *LRRK2* mutation in PD2 patient. While the CRISPR/Cas9 technology has proven extremely beneficial and efficient, it is still hampered by risks related to accuracy and reliability of the system. Apart from targeting the intended mutation, the CRISPRS can also induce off-target effects^46^. To minimize these, we used the Cas9n nickase mutant version of Cas9 that induces a double checkpoint for the target-identification and has been reported to reduce off-target effects by 50–100 folds^47^. Further, we analyzed the expression of the most-likely affected off-target genes and found them unaltered.

## Conclusions

In conclusion, our results show that the PD patient astrocytes manifest several hallmarks of the disease.

**Figure 7:**
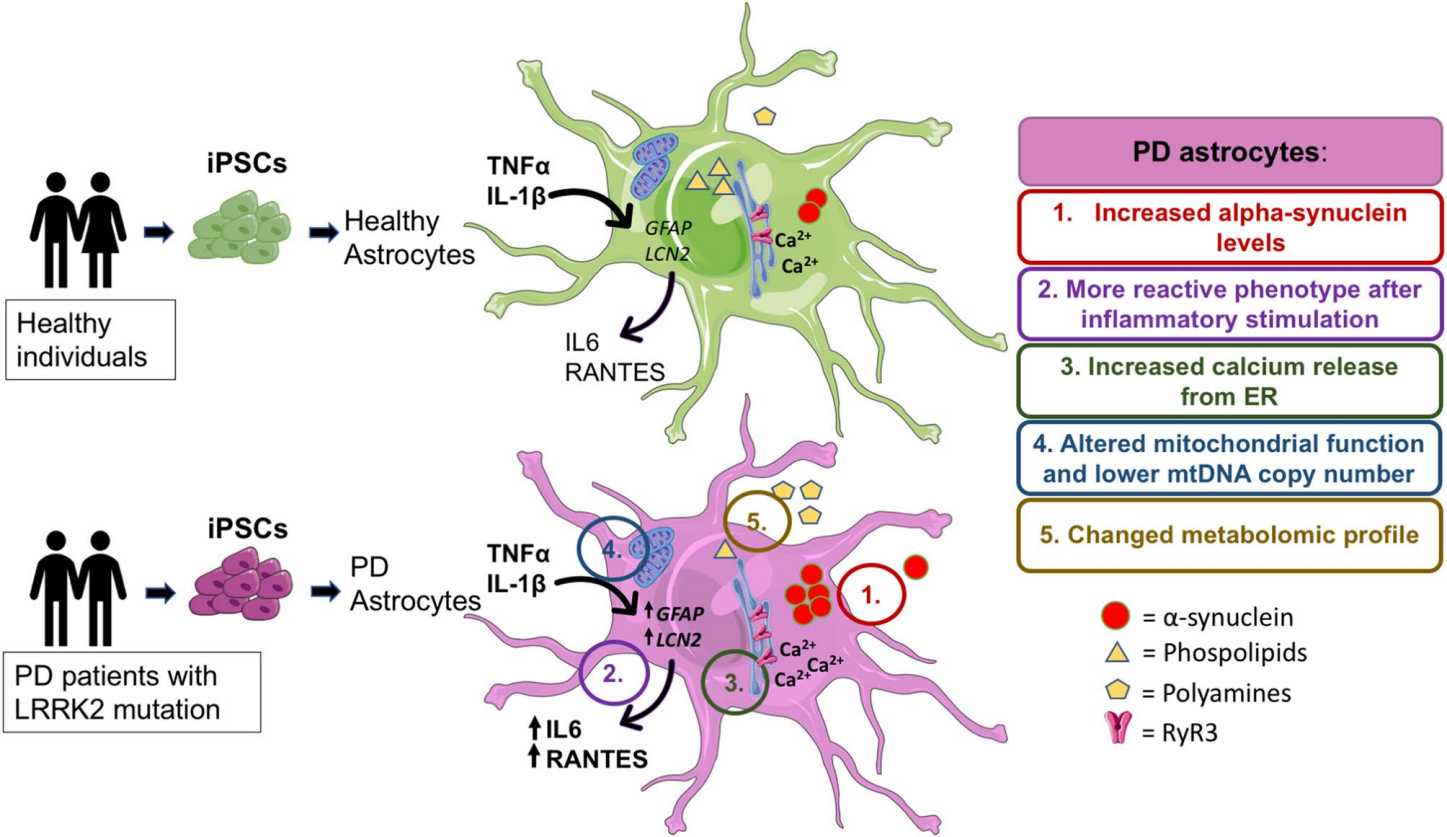
PD patients astrocytes manifest several hallmarks of the disease, these include: (1) increased production of alpha-synuclein, (2) increased reactivity upon inflammatory stimulation, (3) increased astrocytic Ca^2+^ levels, (4) mtDNA maintenance defects and (5) metabolomic changes.

These include increased production of α-synuclein, increased astrocytic Ca^2+^ levels, increased reactivity upon inflammatory stimulation, mtDNA maintenance defects, metabolic changes and altered polyamine metabolism (Fig. 7). Our results provide evidence that *LRRK2* and *GBA* mutant astrocytes are likely to contribute to PD progression and offers new perspectives for understanding the roles of astrocytes in the pathogenesis of PD.

## Methods

### Generation of human iPSCs

The human iPSCs used in this study were generated and characterized in the Stem Cell Laboratory of Molecular Brain Research Group, at the University of Eastern Finland as previously described^16^. The samples were obtained from four individual subjects (Table 1), two of them diagnosed with familial Parkinson’s disease and carrying the G2019S mutation in the *LRRK2* gene. One of the PD subjects carried an additional N370S mutation in the glucocerebrocidase (*GBA)* gene, also a known risk factor for PD (PD1)^48^. Two subjects were healthy controls, with no signs of PD. From each subject two lines were derived and in the majority of experiments 3–4 lines per group has been used.

**Table 1:** Summary of healthy control and PD patients used in this study.

Patient	Sex	Sample collection at age (years)	Genotype	Status	Age at onset	Sample type	Isogenic control line	Reference
Ctrl 1	F	Adult	Normal	Normal		Skin biopsy		Holmqvist et al.^16^
Ctrl 2	M	62	Normal	Normal		Skin biopsy		Holmqvist et al.^16^
PD 1	M	59	LRRK2, GBA	Parkinson’s disease	48	Skin biopsy		Holmqvist et al.^16^
PD 2	M	64	LRRK2	Parkinson’s disease	52	Skin biopsy		Holmqvist et al.^16^
PD 2 iso	M	64	LRRK2 corr	FPD corrected	52	Skin biopsy	LRRK2 (G2019S) corrected	

*iPSC* induced pluripotent stem cell, *Ctrl* control, *F* female, *M* male, *LRRK2* leucine-rich repeat kinase 2, *GBA* acid beta-glucosidase, *FPD* familial Parkinson`s disease.

### Generation of isogenic control lines

Isogenic control line was generated for the PD2 line (Table 1) by correcting the c.6055G>A (G2019S) mutation using CRISPR/Cas9 technology. Specifically, the nicking Cas9 (Cas9n) in combination with 2 single guide RNAs (gRNA) flanking the mutation and a repair template in the form of a ssODN were used (Supplementary Table S1). The LRRK2 specific gRNA sequences were derived using the CRISPRtool (https://tools.genome-engineering.org). Partially complimentary oligonucleotides of the gRNA sequences were cloned either into pSpCas9n(BB)-2A-GFP(PX461; sense guide) or pSpCas9n(BB)-2A-Puro(PX462; antisense guide); these plasmids were a gift from Feng Zhang (Addgene plasmid # 48140 and # 62,987 respectively; https://n2t.net/addgene:48140; RRID:Addgene_48140 and https://n2t.net/addgene:62987; RRID:Addgene_62987^47^). Equimolar amounts of these plasmids and 50 pmol of ssODN-G, the repair template, were co-transfected into the PD2 line using electroporation (Neon electroporator system; 100 µl tips, 1,400 V, 20 ms and 2 pulses; Thermo Fisher Scientific). The transfected cells were then plated on Matrigel (Corning) coated dishes in E8 media (Gibco) with 5 μM ROCK- inhibitor (Sigma) and 0.25 μg/ml puromycin (Gibco)-resistant clones were screened for correction of the *LRRK2* sequence using the Surveyor assay (IDTDNA), according to the manufacturer’s instructions. Briefly, *LRRK2* region spanning the mutation was amplified using Phusion Hot start II enzyme (LifeTechnology) and the primers listed in Supplementary Table S2. Equal amounts of the *LRRK2* amplicon were digested with Nuclease S enzyme. The reaction products were electrophoresed on a 1% agarose gel and visualized using Gel Doc™ XR + (BioRad). Since the parent PD line was heterozygous for the mutation, the lack of digested product indicated correction. Surveyor assay positive clones were sequenced using standard Sanger sequencing (Macrogen Europe) to verify the correction (Supplementary Fig. S1).

### Astroglial differentiation from human iPSCs

Differentiation of astroglial progenitors and astrocytes was based on the publication by Krencik et al.^17^ and modified as previous published by Oksanen et al.^18^. Briefly, iPSCs were seeded on Matrigel-coated plate in E8 with 10 µM ROCK inhibitor. Next day the ROCK inhibitor was removed, and the differentiation was started by changing the E8 medium to neural differentiation medium (NDM) supplemented with 10 µM SB431542 (Sigma) and 200 nM LDN-193189 (Selleckchem). The NDM with the inhibitors was changed every other day until rosettes were formed (days 3 to 10), after that NDM was supplemented with 20 ng/ml of bFGF (Peprotech) for two days to expand the rosettes. The rosettes were picked up and transferred to ULA-plate in astro-differentiation medium where they formed spheres. The spheres were maintained in astro-differentiation medium (consisting of DMEM/F12, 1% N2, 1% Glutamax, 1% non-essential amino acids, 0.5% penicillin/streptomycin (50 IU/50 mg/mL), and 0.5 IU/mL heparin (Leo Pharma) supplemented with 10 ng/mL bFGF and 10 ng/mL EGF (Peprotech) with 10 ng/ml of FGF and EGF (both from Peprotech) and the media were changed three times per week. Spheres were split manually every week and maintained in suspension for 5–8 months. To mature the astrocytes, the spheres were dissociated with accutase (Stem cell technologies) and plated to 12- or 24-well plates in astro-maturation medium (astro-differentiation medium with 10 ng/ml of CNTF and 10 ng/ml of BMP4 (both from Peprotech) for six to seven days with change of media every other day. All the experiments were performed with astrocytes between the age of 6–7 months.

### Glucose uptake

The ability of astrocytes to uptake glucose was analyzed with fluorescent D-glucose analog, 2-(N-(7-Nitrobenz-2-oxa-1,3-diazol-4-yl)Amino)-2-Deoxyglucose (2-NBDG; Thermo Fischer). The cells were seeded on Matrigel-coated 12-well plates and half of the wells were exposed to 2-NBDG, while the rest were used as controls. Prior to exposure to the 2-NBDG solution, the cells were washed with PBS to eliminate glucose from the cells. The cells were incubated in 50 nM 2-NBDG at 37 °C for 30 min. The cells were then washed twice with cold PBS, detached with accutase, collected in glucose-free DMEM (Gibco) and centrifuged. The pellet was washed and resuspended in PBS. The fluorescence was measured by flow cytometry (FACS Calibur) and compared to the non-exposed cells. A proportion of the astrocytes were exposed to 200 nM insulin (Sigma) for 30 min to monitor the effect of insulin on the glucose uptake.

## Glutamate uptake

Astrocytic glutamate uptake was studied with radioactively labeled glutamic acid. The maturated astrocytes were washed with physiologically balanced salt solution (PBS) containing either choline or NaCl and incubated in BSS buffer with choline or NaCl supplemented with 10 µM glutamate (Sigma), and 1 µCi/ml Glutamic Acid L-[3,4-^3^H] (Sigma) for 10 min at 37 °C. After incubation, astrocytes were washed prior to addition of lysis buffer (0.1 M NaOH, 0.01% SDS) on ice. 40 µl of the cell lysis was transferred to a 24-well plate and 600 µl of Ultima Gold was added to the wells. The radioactivity was measured with MicroBeta (Wallac) using 1 min counting time. The results were normalized to protein concentration measured with Pierce BCA protein assay 595 nm kit (Thermo Fisher). The passive glutamate uptake (in presence of choline) was subtracted from sodium-dependent uptake (in presence of NaCl).

### Immunocytochemistry

The cells were fixed with 4% paraformaldehyde for 20 min. For intracellular antigens, the cells were further permeabilized with 0.25% Triton X-100 in PBS (Sigma) for 1 h at room temperature. The cells were blocked with 5% normal goat serum (Vector) in PBS at room temperature (RT) for 1 h and then incubated with primary antibodies in PBS or in blocking buffer at 4 °C overnight. The second day, the cells were incubated in PBS containing a secondary antibody for 1 h at RT in the dark. The cells were washed with PBS, and the nuclei were visualized with DAPI (Sigma, 1:2,000, 5 min). The cells were imaged with Zen Observer Z1 or Zen Imager AX10. The antibodies used in this study are listed in Supplementary Table S2.

### qPCR for mitochondrial copy number determination

To extract the total DNA, the astrocytes were lysed by adding 0.5–1 ml of lysis buffer (lysis solution from GeneJET genomic DNA purification kit, Thermo Scientific) with Proteinase K (Thermo Fisher), harvested and incubated at 55 °C overnight. Next day, an equal volume of isopropanol was added, and the samples were shaken until DNA precipitate became visible. The samples were centrifuged at 12,000 rpm for 5 min to pellet the DNA, which then was washed twice with 70% ethanol. The DNA was dried at room temperature for 15 min, then resuspended in 50 μl of nuclease-free water and incubated at 55 °C for 30 min to dissolve the DNA. The DNA concentration was measured with NanoDrop. The mitochondrial copy number was determined by measuring the relative quantity of mitochondrial *CYTB* gene against a nuclear *APP* gene (Supplementary Table S3) in a SYBR green assay. The qPCR was performed with 25 ng of DNA using the Maxima SYBR green Master Mix (Thermo Scientific).

### qRT-PCR

RNA was extracted using RNA easy MiniKit (Qiagen) according to the manufacture`s protocol. The concentration of the RNA was measured with NanoDrop, and 500 ng of RNA was converted to cDNA. First, 500 ng of RNA was diluted to water, and 1 μl of Random hexamer primer (Fermentas) was added to the samples. The samples were incubated for 5 min at 65 °C in PTC-100 Programmable Thermal Controller (MJ Research inc.). Synthesis mixture (containing: 10 mM dNTP, 5 × reaction buffer for Maxima reverse transcriptase, Ribonuclease inhibitor and Maxima reverse transcriptase (Fermentas) was added to the sample’s and cDNA synthesis was run for 30 min at 50 °C. The qRT-PCR was conducted using Maxima probe/ROX qPCR master mix (Thermo Fisher Scientific) to quantify the relative expression of genes of interest using TaqMan assays (Supplementary Table S4) with Step One Plus (Applied Biosciences). The Ct mean value was normalized to internal Ct mean value of beta actin or GAPDH, and the relative expression is presented as a fold change compared to control group.

### Alpha-synuclein ELISA

To measure the α-synuclein levels from the cells and media, human α-synuclein ELISA kit (Biolegend) was used according to the manufacturer’s instructions. Shortly, the cells were lysed with M-PER lysis buffer and diluted 1 to 20 in reagent diluent. The media samples were not diluted. 200 µl of sample was added to the plate, and the plate was incubated overnight at 4 °C on a shaker. Next day, the plate was washed, and biotinylated primary antibody was added to the plate. After 2 h incubation, the plate was washed and diluted streptavidin HRP was added followed by 1 h incubation. After washes, 100 µl/well of the chemiluminescent substrate was added to the plate, mixed and read with PerkinElmer`s VICTOR multilabel plate reader. The α-synuclein concentration from the cells was standardized to total protein concentration which was obtained using Pierce BCA protein assay 595 nm kit.

### Cytokine exposure and cytometric bead array

Astrocytes were matured for seven days before exposure to TNFα (10 ng/ml), IL-1β (10 ng/ml) or INFγ (10 ng/ml, all fromPeprotech). Medium samples were collected at 24 h time point and analyzed with Cytometric Bead Array (CBA) Human Soluble Protein Master Buffer Kit (BD). First, a set of standards were made according to the manufacturer’s instructions, and 20 µl of the samples and the standards were pipetted to a 96 well plate. Then the capture beads for interleukin 6 (IL-6) and regulated on activation and normal T-cell expressed and secreted (RANTES) were prepared by diluting the beads 1:50 in bead diluent and 20 µl was added to each well followed by one-hour incubation at RT. Next, the PE detection reagents were prepared by diluting the regent 1:50 in reagent diluent and 20 µl was added to the wells. After 2 h incubation in the dark at RT, the samples were washed with 200 µl of washing buffer, centrifuged and vortexed. The samples were resuspended in 100 µl of wash buffer and analyzed with FACSAriaIII (BD Biosciences). At least 300 events per cytokine were measured. Data were analyzed using FCAP Array 2.0 (SoftFlow, Hungary) and cytokine concentrations were calculated by regression analysis from known standard concentrations.

### MitoTracker green

The cells were seeded on Matrigel-coated 12-well plates and half of the wells were exposed to MitoTracker Green (MTG, Life Technologies), while the rest were used as control. The cells were incubated in 250 nM MTG at 37 °C for 20 min. The cells were then washed with PBS, detached with accutase, collected in phenol-free media and centrifuged. The pellet was washed and resuspended in PBS. The fluorescence was measured by flow cytometry (FACS Calibur) and compared to the non-stained cells.

### Seahorse assay

The oxygen consumption rate (OCR) and the extracellular acidification rate (ECAR), indicators of mitochondrial respiration and glycolysis, respectively, were measured in real time from live cells with Seahorse Extracellular Flux (XF) 24 Analyzer (Agilent Technology). In this assay, both OCR and ECAR can be measured simultaneously. Astrocytes were plated on Matrigel-coated XF24 TC plate in the density of 85 000 cells/well in astro-maturation medium and matured for seven days. Before the assay, the medium was changed to Seahorse XF Assay medium (Seahorse DMEM supplemented with 2 mM Glutamax) and incubated for 1 h at 37 °C without CO2 before adding the compounds. The final concentrations of D-glucose and sodium pyruvate were 10 mM and 0.5 mM, respectively. For oligomycin, FCCP, antimycin and rotenone the concentrations were 1.0 μM. The assay was performed according to the manufacturer’s instructions using 2 min mix time, 2 min wait time and 3 min measure cycle. After the assay, the results were normalized to protein concentrations using the Bradford Protein Assay (Biorad). The results were analyzed with Wave program (Agilent Technology), and the wells which did not response were excluded from the final results.

### Ca^2+^ imaging

Calcium (Ca^2+^) signals from astrocytes were recorded with an Olympus FluoView 1,000 confocal microscope (Tokyo, Japan), and data were analyzed as previously described (Oksanen et al. 2017). Cells were loaded with Fluo4 (Fluo-4-acetoxymethyl [AM]-ester, Life Technologies; 5 μM for 20 min). Experiments were carried out at 37 °C. Cells in the recording chamber (Cell MicroControls, Norfolk USA) were perfused with oxygenated (O2 100%) Hank’s balanced salt solution (HBSS) containing 2 mM CaCl_2_. Ca-free 1 mM EGTA-containing HBSS was applied for 3 min for baseline recordings. Cresol (3 mM), a ryanodine receptor (RyR) activator diluted in HBSS was applied directly to the studied area by fast local perfusion system (Cell MicroControls, Norfolk USA). To measure calcium signals, the cells were excited at 488 nm, and the emitted light was collected at 500–600 nm through a × 20 objective. The images were analyzed with FluoView and ImageJ software (https://rsb.info.nih.gov/ij) as previously described^49^. Briefly, fluo‐4 fluorescence intensity is shown as an F/F0 ratio, where F is the background-subtracted fluorescence intensity, and F0 is the background-subtracted minimum fluorescence value measured from each cell at rest.

### Non-targeted metabolite profiling

After the maturation period cells were kept in colorless medium without added growth and maturation factors for one day; the medium was prepared otherwise the same way as the astrocyte differentiation medium. Both medium and cell samples were collected and analysed. Media were collected and preserved while the matured astrocytes (aprox. 1.2 × 10^–6^) were carefully detached from the wells using 16 mm cell scrapers, pelleted by centrifugation for 3 min at 4,000 rpm and stored at – 70 °C. Cell pellets were dissociated with 60 µl of milli-Q-water and then sonicated for 5 min with the GWB 2,200 ultrasonic cleaner to homogenize the cells. 80% methanol was then added in a volume of 240 µl to the cell’s samples. To create reference samples for the ultra-performance liquid chromatography quadrupole time-of-flight mass spectrometry (UHPLC-QTOF-MS) system, a volume of 50 µl was taken from a medium sample corresponding to each cell sample and mixed with 150 µl of 80% methanol. Samples were then sonicated for 5 more min and mixed. Twenty microliters were taken from each cell sample and preserved to run a protein assay for normalization. Finally, samples were centrifuged for 5 min at 13 000 rpm and the supernatant was transferred to HPLC sample vials before running the UHPLC-QTOF-MS analysis. The medium samples were also prepared for UHPLC-QTOF-MS analysis with similar steps. Instrument parameters of UHPLC-QTOF-MS were described in previously published method by Pekkinen et al.^50^. Briefly, the UHPLC-QTOF-MS (Agilent Technologies, Waldbronn, Karlsruhe, Germany) was used to non-targeted metabolite profiling. Hydrophilic and hydrophobic metabolites were separated with reversed-phase (RP) and hydrophilic interaction chromatography (HILIC) combined with positive and negative electrospray ionization (ESI). Raw data from UHPLC-QTOF-MS were filtered in several steps. First, the Agilent Mass Hunter Profinder (Agilent Technologies) software was used to remove noise, identified by the molecular feature extraction algorithm. The remaining data on molecular features were frequency-filtered using Mass Profiler Professional (Agilent Technologies), followed by another run of the molecular feature extraction algorithm. These results were evaluated by manually inspecting the extracted ion chromatogram peaks and removing peaks of low quality. Mass spectrometric data were statistically analyzed with the Mass Profiler Professional software. For the final sets of data, only such molecular features were selected that exhibited higher absolute log2 fold change than 1 of difference in abundance between the control group and the PD group. Identification of the significantly differing metabolites found between the sample groups were generated based on accurate mass and isotope information; i.e., ratios, abundances, and spacing, as well as product ion spectra (MS/MS) by spectral matching to the published databases and laboratory’s own spectral library.

### Statistical analysis

The majority of the data were collected in a blinded manner (ELISA, calcium imaging, metabolomics and ICC) and analyzed using GraphPad Prism (San Diego, CA). The data are presented as mean ± SD and in case of Ca^2+^ as mean ± SEM. The statistical significance was determined with Student’s t-test (upaired and two-tailed between PD1 and PD2) or one-way ANOVA followed by Tukey`s multiple comparisons test (between controls, PD1 and PD2), and p < 0.05 was considered as significant.

### Ethical approval

The study was approved under the ethical approval from the committee on Research Ethics of Northern Savo Hospital district (license no. 42/2010 and 123/2016) obtained by Prof. Jari Koistinaho. Work was carried out according to European and Finnish national rules, with the higest level of ethics.

## Supplementary information

Supplementary Information.

Supplementary Table 7.

## Abbreviations

2-NBDG: 2-(N-(7-Nitrobenz-2-oxa-1,3-diazol-4-yl)amino)-2-deoxyglucose
α-synuclein: Alpha synuclein
AQP4: Aquaporin 4
Cas9n: Nicking Cas9
CI: Complex I
DA: Dopaminergic
ECAR: Extracellular acidification rate
ER: Endoplasmic reticulum
ESI: Electronspray ionization
FCCP: Carbonyl cyanide-p-trifluoromethoxyphenylhydrazone
GBA: Glucocerebrocidase
GFAP: Glial fibrillary acidic protein
GLAST: Glutamate aspartate transporter 1
GLUT1: Glucose transporter 1
GCase: Glucocerebrosidase
gRNA: Guide RNA
HILIC: Hydrophilic interaction chromatography
IL-1β: Interleukin 1 beta
IL-6: Interleukin 6
IFNγ: Interferon gamma
iPSC: Induced pluripotent stem cell
LCN2: Lipocalin-2
LRRK2: Leucine rich repeat kinase 2
MPP+: 1-Methyl-4-phenylpyridinium
MPTP: 1-Methyl-4-phenyl-1,2,3,6-tetrahydropyridine
mtDNA: Mitochondrial DNA
MTG: MitoTracker green
OCR: Oxygen consumption rate
PD: Parkinson’s Disease
RANTES: Regulated on activation and normal T-cell expressed and secreted
RP: Reversed-phase chromatography
RyRs: Ryanodine receptors
S100β: S100 calcium-binding protein beta
SN: Substantia nigra
TNFα: Tumor necrosis factor alpha
UHPLC-QTOF-MS: Ultra-performance liquid chromatography quadrupole time-of-flight mass spectrometry
