# Supplementary material for: Metabolic alterations in Parkinson’s disease astrocytes
Source: Sci Rep. 2020 Sep 2;10:14474. doi: 10.1038/s41598-020-71329-8 (PMC7468111; doi:10.1038/s41598-020-71329-8)
Supplement: Supplementary file 1 — Supplementary Information. [file 41598_2020_71329_MOESM1_ESM.docx]

# Metabolic alterations in Parkinson’s disease astrocytes

Tuuli-Maria Sonninen ^1^, Riikka H Hämäläinen ^1^, Marja Koskuvi ^1^, Minna Oksanen ^1^, Anastasia Shakirzyanova ^1^, Sara Wojciechowski ^1^, Katja Puttonen ^1^, Nikolay Naumenko ^1^, Gundars Goldsteins ^1^, Nihay Laham-Karam ^1^, Marko Lehtonen ^3,4^, Pasi Tavi ^1^, Jari Koistinaho ^1,2^ , Šárka Lehtonen ^1,2*^

^1^A.I.Virtanen Institute for Molecular Sciences, University of Eastern Finland, Kuopio, Finland

^2^ Neuroscience Center, University of Helsinki, Helsinki, Finland

^3^ School of Pharmacy, University of Eastern Finland, Kuopio, Finland

^4^LC-MS Metabolomics Center, Biocenter Kuopio, Kuopio, Finland

# Supplementary material

Table S1: List of oligonucleotides and ssODN

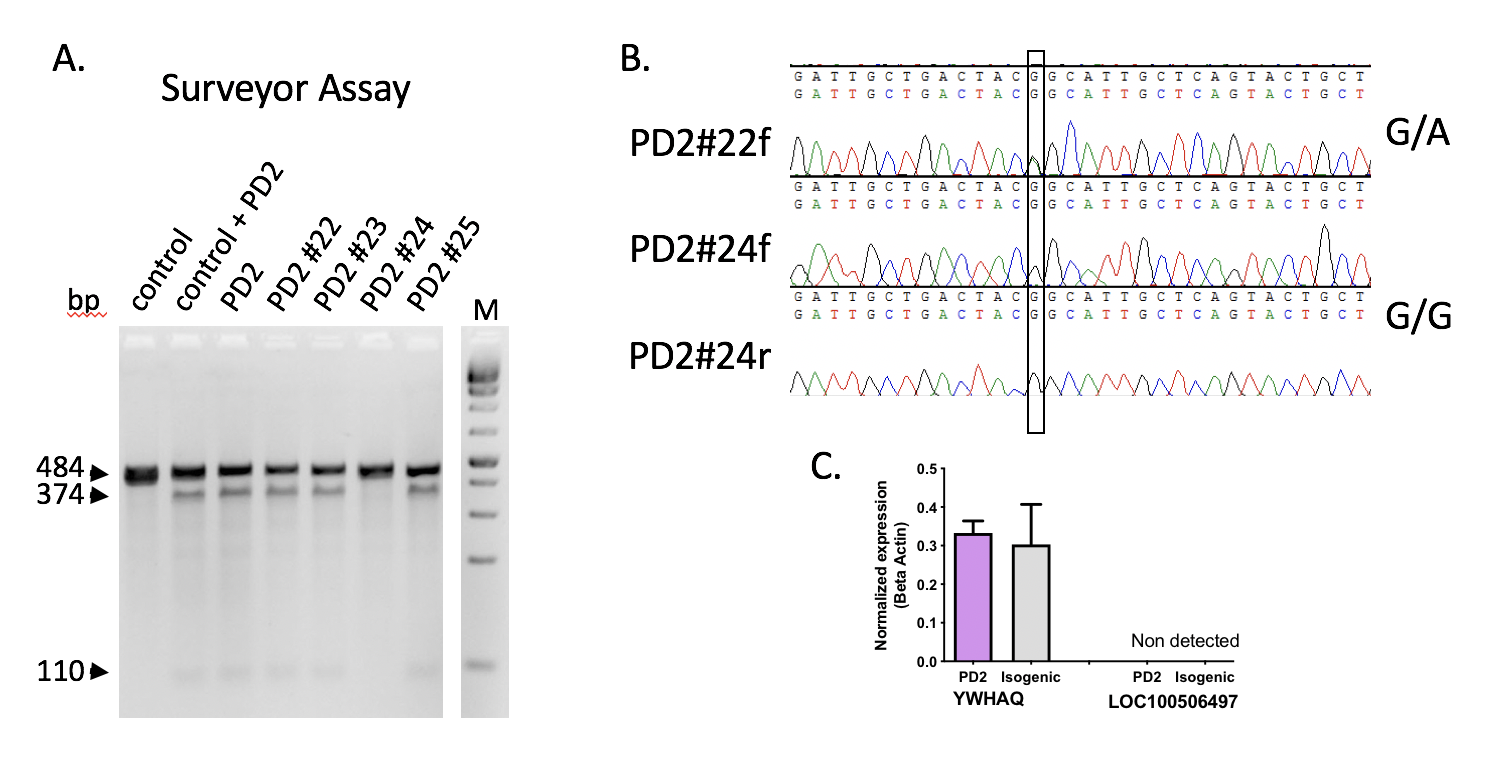


**Figure S1 Derivation of isogenic control for the PD2 iPSC line.**

An isogenic control for the PD2 iPSC line was successfully derived using CRISPR/Cas9 technology. The PD2 parental line was heterozygous for the LRRK2 mutation (c.6055G>A) and this was corrected to the wt sequence. A single clone (PD2#24) from a total of 65 tested clones was positive for the correction. **(A)** A homozygous GC genotype was observed for PD2 clone#24, whereas the other clones were heterozygous GA genotype similarly to the parental line, as demonstrated in the Surveyor assay and **(B)** the correction of the 6055A to G change was confirmed by the Sanger sequences. (C) The RNA expression levels of putative non-specific target genes YWHAQ and LOC100506497 in iPSCs of PD2 and isogenic line.

Table S2. List of antibodies used in immunocytochemistry

| Type | Antibody | Origin | Manufacturer | Product |
| --- | --- | --- | --- | --- |
| Primary | GFAP | mouse | Chemicon | MAB360 |
|  | GFAP | rabbit | Dako | Z0334 |
|  | S100B | rabbit | Swant | 37a |
|  | Alpha-synuclein | mouse | BD Bioscience | 610787 |
|  | Aquaporin 4 | rabbit | Merck | AB3594 |
|  | Vimentin | mouse | Merck | V2258 |
| Secondary | anti-mouse 488 | goat | Molecular Probes | A11001 |
|  | anti-mouse 568 | goat | Molecular Probes | A11004 |
|  | anti-rabbit 488 | goat | Molecular Probes | A11008 |
|  | Anti-rabbit 568 | goat | Molecular Probes | A11011 |

Table S3. Primers for mitochondrial copy number determination.

| **Gene** | **Primer sequences** |
| --- | --- |
| APP | F: GCC TGC CTG ATC CTC CAA AT  R: AGG GTA GCG GAT GAT TCA GCC |
| CYTB | F: TGT GTG CTC TCC CAG GTC TA  R: CAG TTC TGG ATG GTC ACT GG |

Table S4. List of primers used in qRT-PCR

| Gene symbol | Gene name | TaqMan®  Gene Expression Assay ID | |
| --- | --- | --- | --- |
| ACTB | Beta actin | | 4326315 E |
| AQP4 | Aquaporin 4 | | Hs00242342 |
| GFAP | Glial fibrillary acidic protein | | Hs00909233 |
| ITPR2 | Inositol 1,4,5 – trisphosphate receptor,  type 2 | | Hs00181916 |
| LCN2 | Lipocalin-2 | | Hs01008571 |
| LOC10005064 | uncharacterized | | Hs03890527 |
| SLC2A1 | Solute carrier family 2 member 1 | | Hs00892681 |
| SLC1A3 | Solute carrier family 1 member 3 | | Hs00188193 |
| SNCA | Alpha-synuclein | | Hs00240906 |
| YWHAQ | tyrosine 3-monooxygenase/tryptophan 5-monooxygenase activation protein theta | | Hs00863277 |

Table S5 The impact of p-value on the number of features in cells with fold change >2

|  | HILIC (ESI-) | HILIC (ESI+) | RP (ESI-) | RP (ESI+) |
| --- | --- | --- | --- | --- |
| p<0.05 | 47 | 87 | 83 | 67 |
| p<0.01 | 0 | 6 | 27 | 10 |
| p<0.001 | 0 | 0 | 4 | 0 |

Abbreviations: HILIC, hydrophilic interaction liquid chromatography; RP, Reverse-Phase: ESI, electrospray ionization

Table S6 The impact of p-value on the number of features in medium with fold change >2

|  | HILIC (ESI-) | HILIC (ESI+) | RP (ESI-) | RP (ESI+) |
| --- | --- | --- | --- | --- |
| p<0.05 | 49 | 67 | 24 | 39 |
| p<0.01 | 33 | 42 | 16 | 20 |
| p<0.001 | 31 | 34 | 8 | 18 |

Abbreviations: HILIC, hydrophilic interaction liquid chromatography; RP, Reverse-Phase: ESI, electrospray ionization

Table S7. Identified altered metabolites from cells and media of control and PD astrocytes

Attached as separate excel file Table S7.
